# Axl inhibition induces the antitumor immune response which can be further potentiated by PD-1 blockade in the mouse cancer models

**DOI:** 10.18632/oncotarget.21125

**Published:** 2017-09-21

**Authors:** Zhiqiang Guo, Yan Li, Dandan Zhang, Jiaying Ma

**Affiliations:** ^1^ Department of Gynecology and Obstetrics, Shengjing Hospital, China Medical University, ShenYang, China; ^2^ Department of Biotherapy, Cancer Research Institute, The First Affiliated Hospital of China Medical University, ShenYang, China

**Keywords:** Axl, PD-1, immune checkpoint, CD103^+^ DC, R428

## Abstract

Immune checkpoint blockers (ICB) have emerged as a promising new class of antitumor agents which significantly change the treatment landscape in a range of tumors; however, cancer patients benefited from ICB-based immunotherapy remains limited, scoring the need to explore the combination treatments with synergistic mechanisms of action. Axl receptor tyrosine kinase critically involves in the carcinogenesis of multiple cancers due to its dual roles in both promoting cancer invasion and metastasis and suppressing myeloid cell activation and function. Here, we found that Axl inhibition by tyrosine kinase inhibitors induces antitumor efficacy critically depending on immune effector mechanisms in two highly clinical relevant murine tumor models. Mechanistic investigation defined that Axl inhibition reprograms the immunological microenvironment leading to the increased proliferation, activation and effector function of tumor-infiltrating CD4^+^ and CD8^+^ T cells possibly through preferential accumulation and activation of CD103^+^ cross-presenting dendritic cells. More importantly, we show that Axl inhibition induces an adaptive immune resistance evidenced by unregulated PD-L1 expression on tumor cells and combined Axl inhibition with PD-1 blockade mounts a potent synergistic antitumor efficacy leading to tumor eradication. Thus, Axl-directed therapy in Axl expressing tumors could hold a great potential to subvert the innate and/or adaptive resistance to and broaden the coverage of population benefited from ICB-based immunotherapy.

## INTRODUCTION

In recent years, cancer immunotherapy is becoming reinvigorated [1]. In this regard, ICB targeting programmed death-1 (PD-1) and its ligand PD-L1 have produced an unprecedented clinical benefit in cancer patients and been approved by Food and Drug Administration for treating melanoma, Hodgkin lymphoma, Merkel cell carcinoma, head and neck squamous cell carcinoma, non-small cell lung cancer, renal cell carcinoma and urothelial carcinoma with a rapidly growing list of indication [2]. By blocking the negative immune regulatory signals mediated by PD-1/PD-L1 pathway, these drugs remove the inhibition of T-cell activation and effector function and vigorously restore antitumor immune responses [3]. However, PD-1 blockade alone is not able to antagonize all resistance mechanisms and quite a proportion of cancer patients do not respond to this treatment (primary resistance), and some responders relapse after a period of response (acquired resistance) [4]. Therefore, ICB-based combination treatments with synergistic mechanisms of action, that expect to merge individual advantages of immunotherapy and conventional therapies such as chemotherapy, radiotherapy and molecularly targeted therapy, have been avidly proposed and being urgently explored in multiple clinical trials worldwide [4, 5].

The TAM (Tyro3, Axl and Mer) subfamily of receptor tyrosine kinases (RTKs) has been defined to be involved in the carcinogenesis of multiple types of cancer by modulating immune and biology behaviors within tumors [6, 7]. Among them, Axl RTK have been reported to be overexpressed or ectopically expressed in a multitude of cancers which is correlated with increased invasiveness/metastasis, epithelial to mesenchymal transition (EMT) phenotype and drug resistance of cancer cells and poor prognosis of cancer patients [8, 9]. For example, Axl and its ligand Growth arrest specific gene 6 (Gas6) have been described to be frequently overexpressed in epithelial ovarian cancer, especially in advanced high-grade serous and endometrioid metastatic ovarian cancer [10–12], and Axl expression is correlated with tumor metastasis, chemoresistance and poor prognosis [11–14]. Targeting Axl in animal tumor models with small molecule tyrosine kinase inhibitors (TKIs), monoclonal antibodies (mAb) or decoy fusion protein have been demonstrated to be able to inactivate Axl-mediated signaling pathways leading to metastasis inhibition, recovered drug sensitivity and improved therapeutic efficacy, defining Axl as a promising novel target for cancer therapeutics [9, 15, 16]. Specifically, Axl has been also demonstrated to be constitutively or inducibly expressed on multiple immune cells, particularly dendritic cells (DCs) and macrophages, which act as an intrinsic negative feedback rheostat to balance the proinflammatory signaling derived from Toll-like receptor and cytokine receptor [17, 18]. Intriguingly, Aguilera et al. recently demonstrated that Axl is an essential gene expressed in mouse tumors refractory to combinatorial irradiation and immunotherapy (anti-PD-1 and/or anti-CTLA4) and its knockdown reprograms the immunological microenvironment leading to tumor sensitization [19]; furthermore, Hugo et al. reported that Axl is a key gene expressed in tumors from patients innately resistant to anti-PD-1 immunotherapy through analysis of the somatic mutanomes and transcriptomes of pretreatment melanoma biopsies [20], indicating that Axl may modulate innate immune cells to dampen the adaptive antitumor immune responses reinvigorated by anti-PD-1 immunotherapy other than its intrinsic protumor roles in tumor cells. Therefore, disabling Axl signaling may promote engagement of adaptive immunity and complement ICB-based immunotherapy.

In view of protumor roles of Axl via acting on both immune cells and tumor cells, herein, we sought to explore whether Axl inhibition would elicit the antitumor immune effects and, if do, whether it would produce a synergy with anti-PD-1 immunotherapy with complementary mechanisms of action in mouse tumor models.

## RESULTS

### Mouse cancer cells express Axl which recapitulates the biological function of their human counterparts *in vitro*

**Figure 1:**
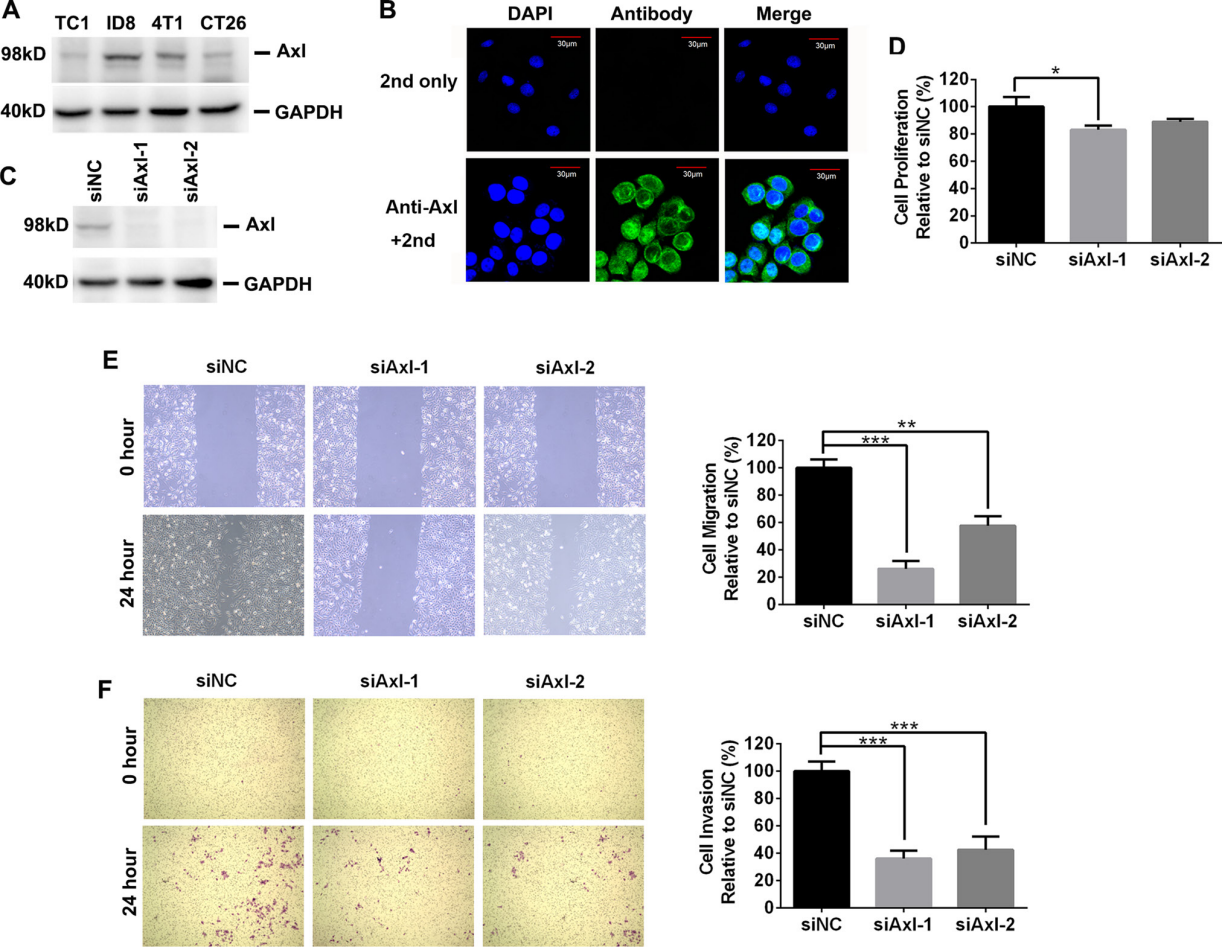
The expression and biological role of mouse Axl in mouse tumor cells (**A**) Axl expression in 4 mouse tumor cell lines was determined by Western blotting. Data are representative of two independent experiments. (**B**) Axl expression in ID8 cells was evaluated by immunofluoresecent staining. Up and bottom pictures display the control (only 2nd staining) and Axl staining respectively. 2nd: secondary antibody; Scale bar, 30 μm. (**C**) Efficient knockdown of Axl expression in ID8 cells transfected with control (siNC) or siRNA specific for Axl (siAxl-1 and siAxl-2). (**D**) The proliferation of ID8 cells with or without Axl knockdown. Proliferation was plotted as a percentage of growth relative to control siNC-treated cells. (**E**) ID8 cells with or without Axl knockdown were subjected to wound exposure. Wound length was imaged and measured after the wound was first made (0 hour) and 24 hours post wound exposure with statistics and representative images (10× magnification) shown. Migration was plotted as a percentage relative to zero time point of each treated cells. (**F**) ID8 cells with or without Axl knockdown were subjected to invasion assay with statistics and representative images (10× magnification) shown. Invasion was plotted as a percentage relative to control siNC-treated cells. The experiments were performed twice with 3 technical replicates, and data are expressed as mean ± SEM (for D–F), *p < 0.05, **p < 0.01, ***p < 0.001, one-way ANOVA followed by Tukey's multiple comparisons test.

We first determined the expression of Axl in 4 murine tumor cells (ID8 ovarian cancer, TC1 lung cancer, 4T1 breast cancer and CT26 colorectal cancer cells) by Western blotting. As shown in Figure 1A, we detected the expression of Axl in all murine tumor cells, consistent with the expected size (98 kD) of mouse Axl protein. The Axl expression in these murine tumor cells was also confirmed by fluorescence-activated cell sorting (FACS) analysis (Supplementary Figure 1). Notably, ID8 and 4T1 cells express a higher level of Axl than TC1 and CT26 cells. The Axl expression in ID8 cells was further validated by immunofluorescence where Axl staining clearly marked the cell surface and cytoplasm consistent with the location of Axl expression (Figure 1B). We did not detect the expression of Mer, another member of TAM RTKs, by FACS analysis (data not shown).

Previous studies demonstrated that Axl expression in human ovarian cancer cells promoted their migration and invasion [11]. To probe the clinical relevance of mouse Axl expression in ID8 cells, we knocked down the mouse Axl expression in ID8 cells by transient transfection of small interference RNA (siRNA) and then evaluated their proliferation, migration and invasion. Western blotting confirmed the specificity and efficiency of siRNA-mediated Axl knockdown as Axl-specific siRNA (siAxl-1 or siAxl-2) but not non-targeting siRNA control (siNC) significantly silenced the Axl expression in ID8 cells (Figure 1C). Though genetic ablation of Axl with either siAxl-1 or siAxl-2 only exhibit the moderate inhibitory effect on the proliferation of ID8 cells (Figure 1D), it markedly inhibit their migration and invasion (Figure 1E–1F). Collectively, these data indicated that ID8 cells expressed Axl and were dependent on this receptor for migration and invasion, which is reminiscent of human ovarian cancer cells.

**Figure 2:**
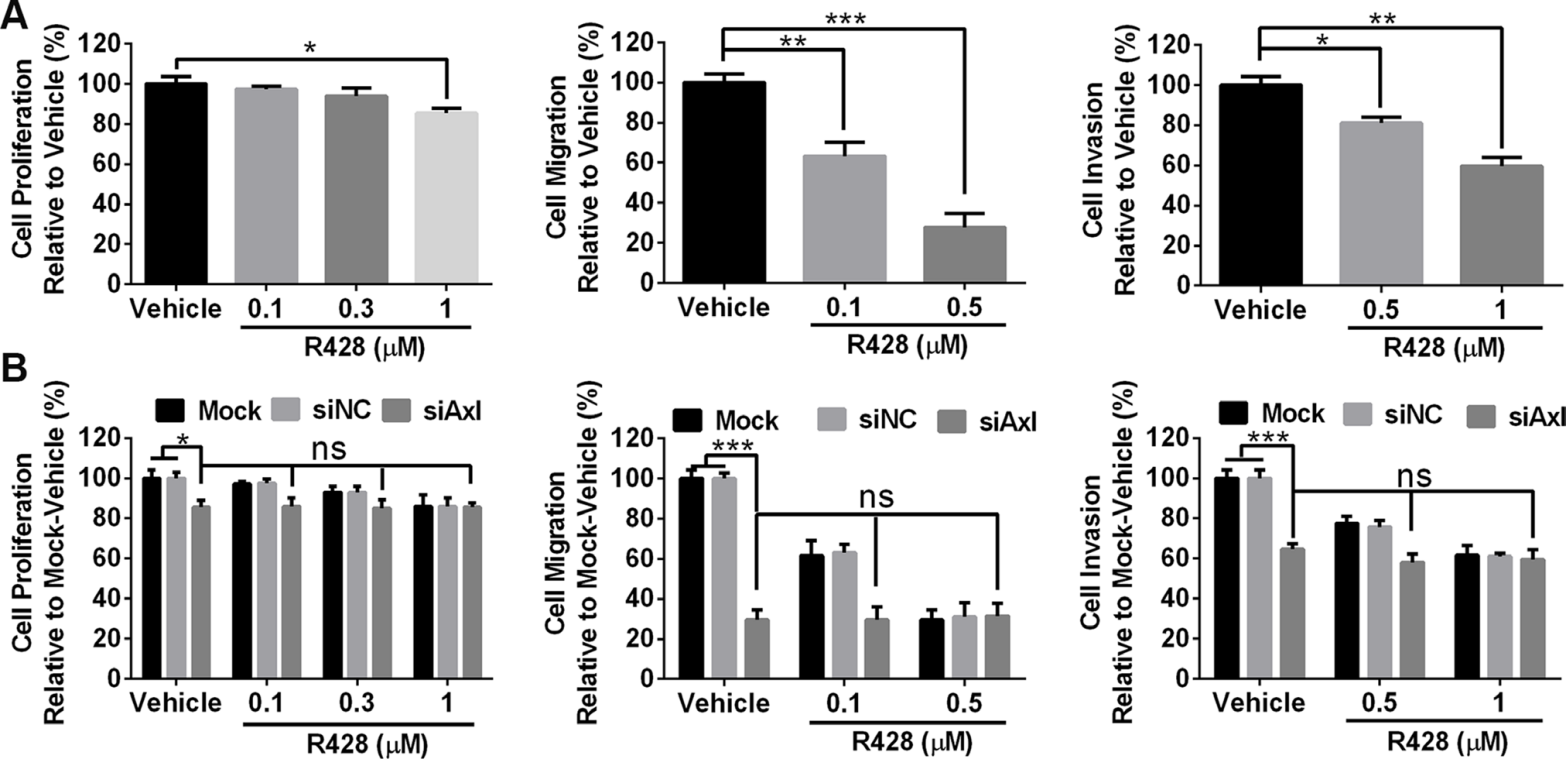
Axl-targeting inhibitor R428 inhibits the proliferation, migration and invasion of mouse tumor cells (**A**) ID8 cells were treated with vehicle or R428 at indicated doses before performing proliferation, migration and invasion assays. (**B**) ID8 cells were transfected with mock, control siNC or siRNA specific for Axl (siAxl-2) and subsequently subjected to proliferation, migration and invasion assays. Proliferation and invasion was plotted as a percentage of growth relative to vehicle-treated cells with migration plotted as a percentage relative to zero time point of each treated cells. The experiments were performed thrice with 3 technical replicates, and data are expressed as mean ± SEM, ns, not significant, *p < 0.05, **p < 0.01, ***p < 0.001, one-way ANOVA followed by Tukey's multiple comparisons test.

Since ID8 cells are sensitive to Axl knockdown by siRNA, we hypothesized that Axl tyrosine kinase inhibitor R428 performs a similar effect. R428 specificity for Axl has been previously evaluated [21], and this agent has now undergone successful phase Ia clinical evaluation [22]. ID8 cells were treated with increasing doses of R428 (0.1–1.0 uM) for 72 hours prior to performing proliferation, migration and invasion assays. Consistent with the results from siRNA assays above, R428 significantly inhibited the migration and invasion of ID8 cells with modest effect on proliferation (Figure 2A). We also determined the inhibitory effect of R428 in mock-transfected, control (siNC) or Axl-silenced (siAxl) ID8 cells; as shown in Figure 2B, R428 exhibited the almost same effect in mock-transfected or siNC cells as in wild-type cells; however, no inhibitory effect for migration and invasion was seen in Axl-silenced cells when they were treated with R428, validating that R428 executes the inhibitory effect by acting on Axl.

As described above, murine 4T1 breast cancer cells also had a high level of Axl expression where suppression of its expression or activity by RNA knockdown or R428 similarly abated their migration and invasion *in vitro* (Supplementary Figure 2). Overall, murine tumor cells express Axl which functions in a similar metastasis-promoting effect as that of human counterpart, suggesting that murine tumor cells such as ID8 may represent a suitable tumor model to examine the potential intervening effect of novel Axl-targeting therapeutics.

### Targeting Axl inhibits tumor growth partially depending on the immune system

**Figure 3:**
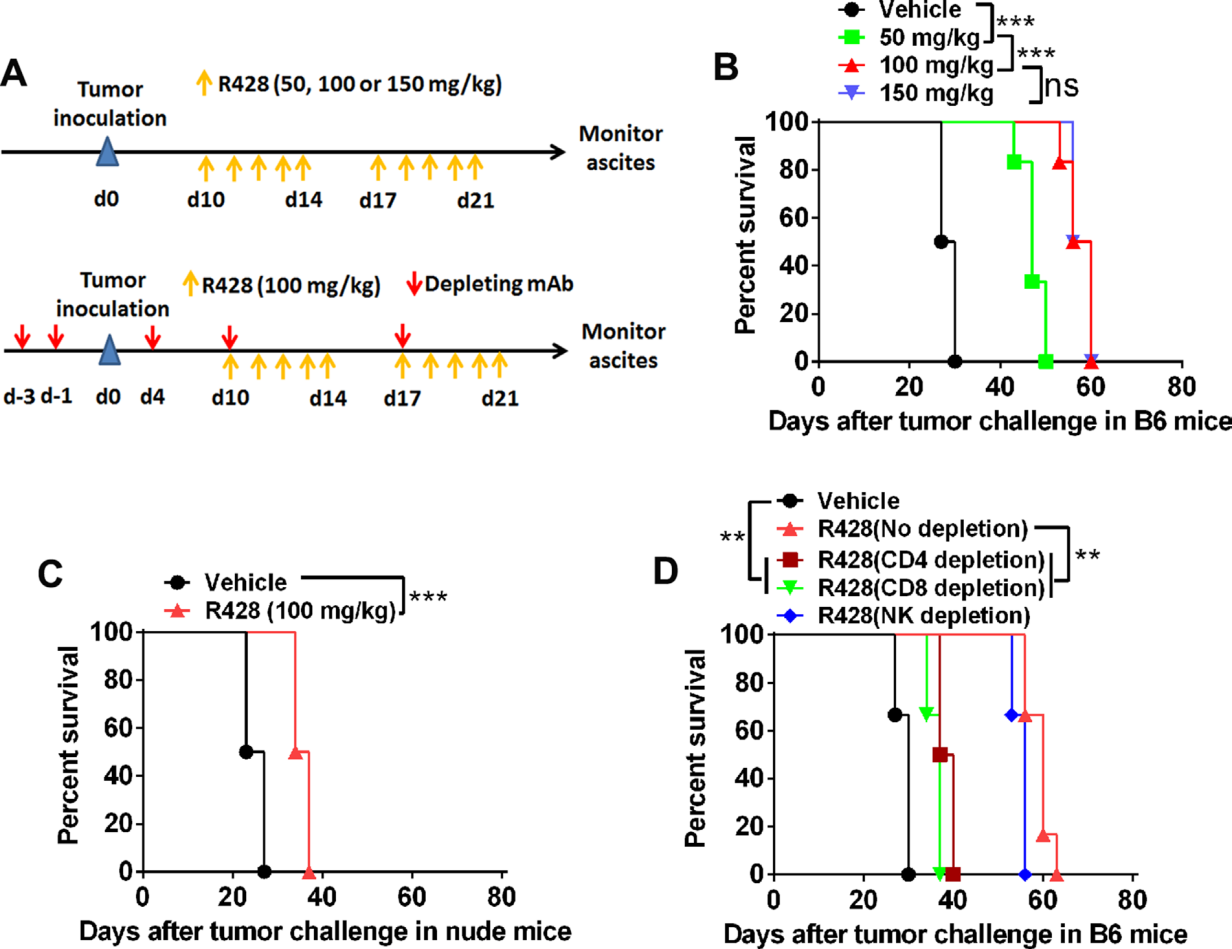
Targeting Axl inhibits tumor growth partially depending on the immune system (**A**) Schematic regimen for testing the *in vivo* optimal dose of R428 (top) or R428 treatment in mice depleted of lymphocyte subsets (bottom). (**B**) C57BL/6 mice (6 per group) bearing 10-day-established ID8 tumors were treated with control vehicle or R428 at the indicated dose for two weeks and their overall survival was evaluated. (**C**) Nude mice (6 per group) bearing 10-day-established ID8 tumors were treated with R428 at the indicated dose for two weeks and their overall survival was evaluated. (**D**) C57BL/6 mice (6 per group) bearing 10-day-established ID8 tumors with lymphocyte subset depletion were treated with R428 at the indicated dose for two weeks and their overall survival was evaluated. Data are representative of two (B and D) or three (C) independent experiments, **p < 0.01, ***p < 0.001, log-rank test (B–D).

Axl inhibition by small molecule inhibitors, receptor fusion protein antagonist or RNA knockdown has been reported to prevent tumor progression in the xenograft model of human cancer [8, 9]. To test whether R428, a well-characterized Axl-specific small molecule inhibitor, can exhibit the tumor-suppressing activity in the progressing ID8 tumor *in vivo*, we treated C57BL/6 mice bearing 10-day-established ID8 tumors with escalating doses of R428 and evaluated antitumor effects defined as the overall survival of tumor-bearing animals as previously described [23]. We first tentatively tested 3 different dose regimens to determine the optimal dose for R428 administration in this tumor model as previous studies reported (see ref [21]; Figure 3A, upper). Figure 3B shows that R428 treatment with the dose of 50 mg/kg for 2 weeks significantly prolonged the overall survival of tumor-bearing mice (median survival time (MST) for vehicle vs 50 mg/kg R428: 28.5 vs 41.5 days, *p* = 0.009). Dose escalation further increased the antitumor efficacy where the dose of 100 mg/kg came to the optimum (MST for 50 mg/kg vs 100 mg/kg R428: 41.5 vs 46 days, *p* = 0.0087). A further dose escalation did not translate into better antitumor efficacy (MST for 100 mg/kg vs 150 mg/kg R428: 46 vs 46 days, not significant). Notably, through regular monitoring of animal's weight, we did not observe obvious toxic effects in mice receiving R428 treatment (data not shown). Thus, we selected 100 mg/kg for 2 weeks as an optimal dose schedule in the following experiments.

To determine whether R428 treatment induces an immune effect to prevent tumor growth *in vivo*, we treated nude mice bearing 10-day-established ID8 tumors with the optimal dose of R428 described above. Unexpectedly, the tumor-suppressive effect of R428 sharply decreased as compared to that observed in syngeneic mice with median survival time of 35.5 days from R428-treated ID8 tumor-bearing nude mice (Figure 3C), suggesting that R428 may suppress tumor growth by indirectly engaging immune system except for directly acting on tumor cells. To confirm this hypothesis, we repeated the above experiments in syngeneic C57BL/6 mice that were depleted of lymphocyte subsets, including CD4^+^T cells, CD8^+^T cells or natural killer (NK) cells (Figure 3A, bottom). As shown in Figure 3D, removal of CD4^+^T cells or CD8^+^T cells significantly weakened the antitumor effect of *in vivo* R428 treatment, indicating that R428 may engage the immune effectors to inhibit tumor growth *in vivo*. Depletion of NK cells also slightly reduced the antitumor effect which is, however, not significant. We obtained the similar results when testing R428 in the murine 4T1 breast cancer model (Supplementary Figure 3).

To test whether immune effect by Axl targeting is unique to R428 treatment, we evaluated the antitumor effect of another Axl-specific inhibitor SGI-7079 which previously demonstrated to specifically inhibit Axl activity [24]. *In vitro* SGI-7079 treatment similarly inhibited the migration and invasion of Axl-sufficient (Mock or siNC) but not Axl-silenced (siAxl) ID8 cells (Supplementary Figure 4A and 4B), validating that SGI-7079 targets to Axl for biological effects. As expected, we also observed that *in vivo* SGI-7079 treatment produced a greater antitumor effect in syngeneic C57BL/6 mice (median survival time 56 days) than that seen in nude mice (median survival time 35.5 days; Supplementary Figure 4C and 4D), indicating antitumor immune effects induced by Axl targeting is not drug-specific.

### Axl inhibition remodels the tumor microenvironment toward immune stimulation

**Figure 4:**
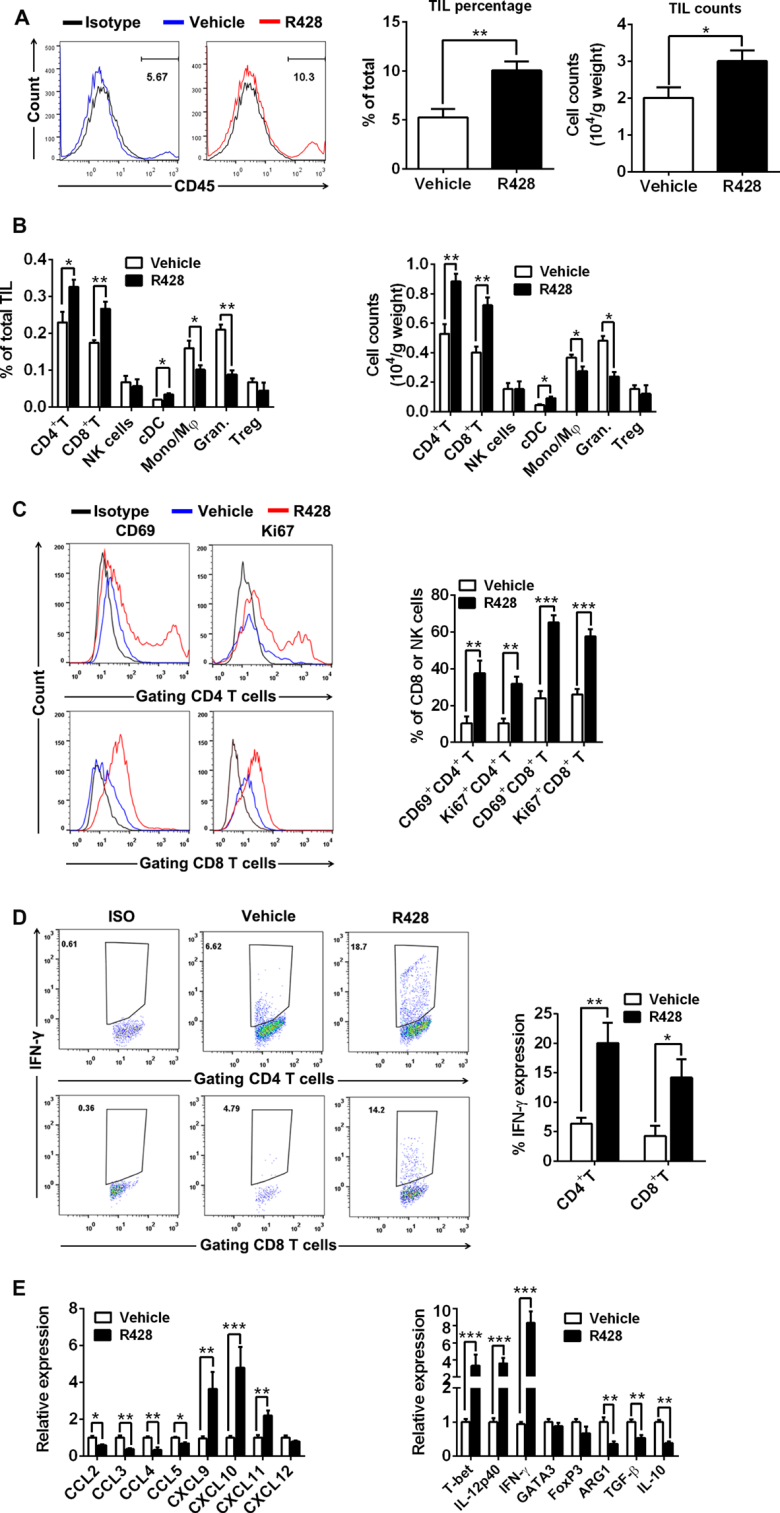
Immune effector mechanisms induced by Axl inhibition C57BL/6 mice (3 per group) bearing 10-day-established ID8 tumors were treated with control vehicle or R428 for 5 days and then TILs and immune-associated genes within tumors were analyzed by FACS and qPCR respectively. (**A**) The representative FACS plots, the percentage and absolute number of CD45^+^ TILs in tumors from vehicle or R428 treated mice. (**B**) The percentage and absolute number of CD4^+^ FoxP3^–^ T cells, CD8^+^ T cells, CD3^+^ DX5^+^ NK cells, CD11c^+^ MHCII^+^ cDCs, CD11b^+^ F4/80^+^ Ly-6G^–^ monocytes/macrophages (Mono/Mφ), CD11b^+^ F4/80^–^ Ly-6G^+^ granulocytes and CD4^+^FoxP3^+^ Treg cells within TILs. (**C**) The percentages and representative FACS plots of CD69^+^ and Ki-67^+^ cells among tumor-infiltrating CD4^+^ T cells and CD8^+^ T cells. (**D**) The percentages and representative FACS plots of IFN-γ^+^ cells among tumor-infiltrating CD4^+^ T cells and CD8^+^ T cells in response to phorbol myristyl acetate (PMA)/Ionomycin stimulation. (**E**) The expression of genes associated with type-1 T cell or suppressive cells recruitment and functionality. Mean values of messenger RNA levels in control group were set to 1. Data are derived from three tumors per treatment and representative of two independent experiments, *p < 0.05, **p < 0.01, ***p < 0.001, two-tailed student *t* test.

To analyze the immune effects induced by Axl inhibition, we evaluated the tumor masses from R428-treated tumor-bearing mice for the composition of tumor-infiltrating lymphocytes (TILs) and the expression of immune-associated genes by FACS and quantitative PCR (qPCR) respectively. TIL analysis revealed that R428 treatment greatly increased the infiltration (percentage and number) of total leukocytes (Figure 4A), among which the percentage and absolute number of CD4^+^ T cells, CD8^+^ T cells and conventional CD11c^+^MHC-II^+^ DCs (cDCs) significantly increased and of granulocyte and monocyte/macrophages significantly decreased (Figure 4B) with no marked change in the compartments of NK cells and regulatory T (Treg) cells. Additional phenotypic and functional analysis showed that CD4^+^ T cells and CD8^+^ T cells from R428-treated tumors had a high proportion of CD69 and Ki67 expression and IFN-γ production (Figure 4C–4D), indicating their increased activation, proliferation and effector function. In agreement with the result of FACS analysis, qPCR assay demonstrated that R428 treatment markedly enhanced the gene expression associated with type-1 T cell recruitment (i.e., increased transcripts for chemokine CXCL9, CXCL10 and CXCL11) and functionality (i.e., increased transcripts for IFN-γ, IL-12p40 and T-bet; Figure 4E) while decreasing gene expression related with suppressive cell recruitment (i.e., reduced transcripts for CCL2, CCL3, CCL4 and CCL5) and function (i.e., decreased transcripts for Arginase-1 (ARG1), TGF-β and IL-10; Figure 4E). We also observed the similar results in the murine ID8 tumor treated with SGI-7079 (data not shown) or murine 4T1 tumor treated with R428 (Supplementary Figure 5). The results support an induction of tumor immunological microenvironment toward stimulatory antitumor immune response by Axl inhibition.

### Axl inhibition promotes accumulation and activation of CD103^+^ cDCs within tumors

**Figure 5:**
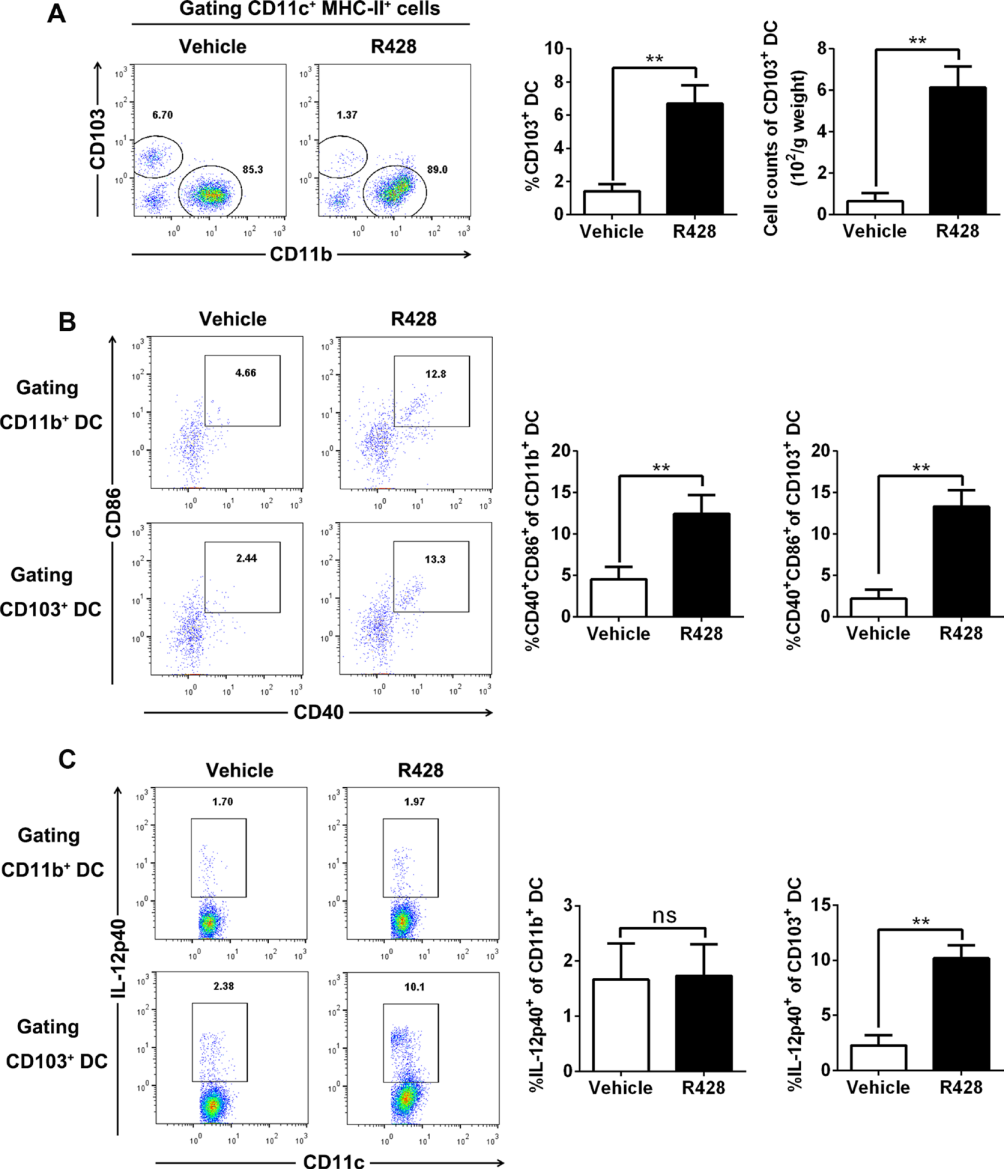
Axl inhibition promotes CD103^+^ DC accumulation and activation C57BL/6 mice (3 per group) bearing 10-day-established ID8 tumors were treated with control vehicle or R428 for 5 days and tumor-infiltrating cDCs were analyzed by FACS. (**A**) Representative FACS plots for CD103 versus CD11b within CD11c^+^ MHCII^+^ cDC gate, and percentage (within CD11c^+^ MHCII^+^ cells) and absolute number of CD103^+^ CD11c^+^ MHCII^+^ cDCs. (**B**) Representative FACS plots for CD86 versus CD40 within CD11b^+^ or CD103^+^ CD11c^+^ MHCII^+^ gate and percentage of CD86^+^ CD40^+^ within CD11b^+^ or CD103^+^ CD11c^+^ MHCII^+^ cDCs. (**C**) Representative FACS plots for CD11c versus IL-12p40 within CD11b^+^ or CD103^+^ CD11c^+^ MHCII^+^ gate and percentage of IL-12p40^+^ within CD11b^+^ or CD103^+^ CD11c^+^ MHCII^+^ cDCs. Data are representative of two independent experiments, *p < 0.05, **p < 0.01, two-tailed student *t* test.

Previous studies show that Batf3-dependent CD8a^+^ and/or CD103^+^ cDCs play an essential role in antitumor immunity which can exploit Axl activation as an intrinsic negative feedback to inhibit ongoing immune response [17, 25–28], we therefore assessed the impact of Axl inhibition on the prevalence and activation status of cDCs at the tumor site, focusing on the CD103^+^ subset. As shown in the above results, Axl inhibition by R428 treatment significantly increased the percentage and absolute number of tumor-infiltrating cDCs (Figure 4B); this increase was predominantly derived from CD103^+^ cDCs when we further divided those cDC into two subsets of CD103^+^ and CD11b^+^ cells (Figure 5A). In addition, both CD103^+^ cDCs and CD103^–^ CD11b^+^ cDCs expressed higher levels of costimulatory molecules in R428-treated tumors (Figure 5B). Finally, the fraction of intratumoral CD103^+^ cDCs producing IL-12 p40 was significantly higher in R428-treated tumors (Figure 5C). Thus, Axl inhibition enhanced the accumulation and activation of Batf3-dependent CD103^+^ cDCs within tumors which may underlie the antitumor effects of Axl inhibition.

### Axl inhibition activates PD-1/PD-L1 pathway whose blockade potentiates the antitumor effect

**Figure 6:**
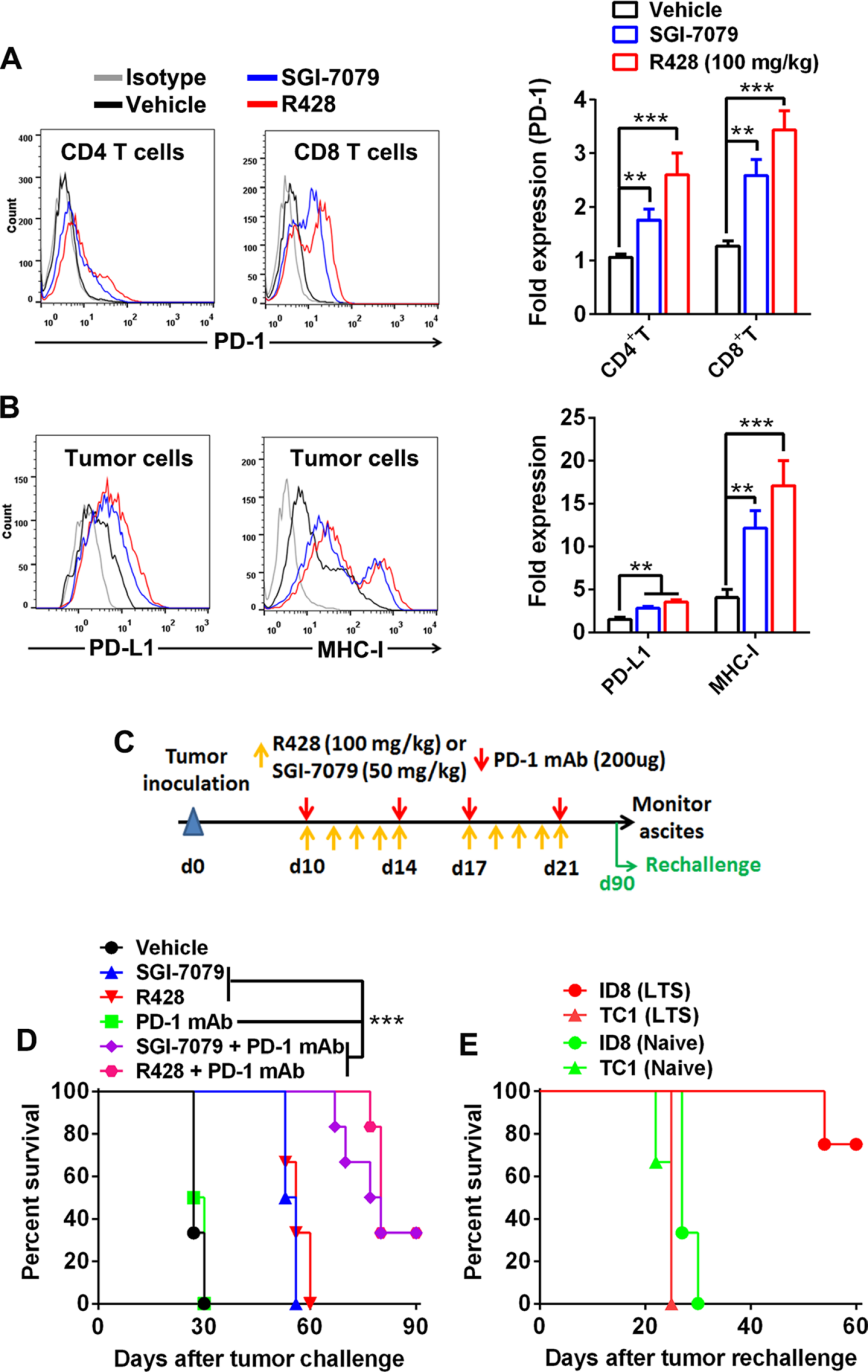
Axl inhibition activates PD-1/PD-L1 pathway and produces a synergistic antitumor effect with PD-1 blockade (**A**–**B**) C57BL/6 mice (3 per group) bearing 10-day-established ID8 tumors were treated with control vehicle, R428 or SGI-7079 for 5 days and tumor cells and tumor-infiltrating T cells were analyzed by FACS. (A) The representative FACS plots (left) and statistical results (right) of PD-1 expression on tumor-infiltrating CD4^+^ T cells and CD8^+^ T cells. (B) The representative FACS plots (left) and statistical results (right) of PD-L1 and MHC-I expression on tumor cells. (**C**) Schematic regimen for combined Axl inhibition (R428 or SGI-7079 treatment) and PD-1 blockade in ID8 tumor-bearing mice. (**D**) C57BL/6 mice (6 per group) bearing 10-day-established ID8 tumors were treated with either single or combined R428/SGI-7079 and anti-PD-1 mAb for two weeks and their overall survival was evaluated. (**E**) Ninety days after first tumor challenge, long-term survivors (LTS) were rechallenged with ID8 (i.p.) or unrelated TC1 (s.c.) tumor cells and their tumor growth was evaluated. Naïve mice with same challenge were used as controls. Data are representative of two (A and B) or three (D) independent experiments, **p < 0.01, ***p < 0.001, one-way ANOVA followed by Tukey's multiple comparisons test (A and B) or log-rank test (D).

Though Axl inhibition alone produced a prominent tumor-suppressing effect, all mice succumb to the death at the end of experiments; we then determined the expression of PD-1/PD-L1 pathway molecule within tumors since this immune checkpoint molecule pathway play a pivotal role in mediating an adaptive immune resistance. FACS analysis demonstrated that tumor-infiltrating CD8^+^ T cells from tumors subjected to Axl inhibition by either R428 or SGI-7079 had a prominent induction of PD-1 expression on their surface, consistent with the increased activation phenotype of these cells described above (Figure 6A); PD-1 expression on tumor-infiltrating CD4^+^ T cells also increased after Axl inhibition whose amount, however, remains low in comparison to CD8^+^ cells. In addition, we observed the significantly increased expression of PD-1 ligand PD-L1 as well as MHC-I molecules on tumor cells from those treated tumors, consistent with the increased IFN-γ production from them (Figure 6B). Based on these observations, we then evaluated whether Axl inhibition would synergize with blockade of PD-1/PD-L1 pathway to elicit a stronger antitumor immune response, we treated C57BL/6 mice bearing 10-day-established ID8 tumors with either anti-PD-1 mAb, SGI-7079 or R428 alone or in combination. As shown in Figure 6C and 6D, combined treatment of Axl inhibition and PD-1 blockade resulted in the cure of one third of treated mice, and a delay of tumor growth and prolongation of their survival in the remaining mice (median survival time for vehicle, SGI-7079, R428, anti-PD-1, SGI-7079/anti-PD-1 and R428/anti-PD-1: 27, 54.5, 56, 78.5 days and not reached) while single treatment with anti-PD1 mAb showed negligible antitumor efficacy. We also observed a similar antitumor effect in the murine 4T1 breast cancer model (Supplementary Figure 6A). Importantly, we did not note any apparent adverse effects in animals treated with combined Axl inhibition and PD-1 blockade (Supplementary Figure 6B).

To test whether combined treatment induced a long-term immune memory, we rechallenged 6 cured mice from initial combined treatment with the same ID8 tumor cells (4 mice) or with the unrelated TC1 lung cancer cells (2 mice). Those long-term surviving mice were resistant to the rechallenge with ID8 tumor with 3 of 4 mice surviving for more than 60 days without any sign of ascites formation though they were susceptible to the outgrowth of unrelated TC1 tumor (Figure 6E). As a control, naïve mice had progressive tumor growth when inoculated with either ID8 or TC1 tumor cells (Figure 6E). The result indicates that mice cured by combined treatment mounted a systemic long-term memory immune response to tumor antigens expressed specifically in the ID8 tumor.

## DISCUSSION

With the expanding role of ICB-based immunotherapies and their clinical benefits, much attention has been paid on the immune system and its potential to eradicate tumor [2]. Consequently, there is renewed interest to explore how commonly used and/or emerging novel anti-cancer modalities have an impact on antitumor immune response [29]. In the present study, out findings demonstrate that inhibition of Axl, a frequently overexpressed functional RTK in many types of cancer involving in tumor malignancy and immune modulation, triggers the anti-cancer efficacy critically depending on tumor cell-extrinsic immune effector mechanisms in two highly clinical relevant murine tumor models. Further mechanistic investigation defined that Axl inhibition reprograms the immunological microenvironment leading to the increased proliferation, activation and effector function of tumor-infiltrating CD4^+^ and CD8^+^T cells possibly through preferential accumulation and activation of CD103^+^ cDCs, which is in favor of antitumor immune response. More importantly, we show that Axl inhibition leads to adaptive immune resistance evidenced by PD-L1 and its receptor PD-1 expression on tumor cells and tumor-infiltrating T cells respectively and thus provides a strong rationale for combination treatment with PD-1 blockade that was supported by a potent synergistic antitumor efficacy derived from combination treatment of Axl inhibition and anti-PD-1 mAb.

Accumulating evidence has defined TAM RTKs as major therapeutic targets due to its critical role in carcinogenesis [7, 9, 16]. TAM RTKs, particularly Axl and Mer, play important roles not only in tumor invasion and metastasis and therapeutic resistance but also in fostering an immunosuppressive microenvironment [7, 19]. Axl has been shown to thwart DC activation and along with Mer decrease NK cell antitumour activity promoting cancer metastasis [17, 18, 30]. Meanwhile, Mer is a critical component of the efferocytosis machinery responsible for clearance of dead cells to maintain tissue homeostasis, and its engagement promotes macrophage release of the anti-inflammatory cytokines IL-10 and TGF-β, polarizing them toward anti-inflammatory M2 phenotype [7, 19, 31]. Thus, Axl and Mer complementarily participate in key steps of antitumor immunity through negative regulation of DCs, macrophages and NK cell activity and induction of immunosuppressive mediators collectively favoring the escape of tumor cells, which makes them as an emerging class of innate immune checkpoints [6]. Supporting this concept, our findings clearly demonstrates that Axl inhibition by either R428 or SGI-7079, two differently designed small-molecule TKIs [21, 24], engaged immune effector arms to inhibit tumor progression as evidenced by prominently increased antitumor efficacy in syngeneic mice versus immunodeficient nude mice as well as decreased antitumor efficacy in syngeneic mice depleted of T cells. One caveat should be noted that both R428 and SGI-7079 also display an inhibitory activity on two other members of TAM RTKs, Mer and Tyro3, with a comparatively higher IC50 value [21, 24]; though our *in vitro* studies show that either R428 or SGI-7079 treatment had little effects in Axl-deficient tumor cells, it remains to be unable to completely exclude that they mount antitumor immune response by accidently suppressing the activity of Mer/Tyro3 and even other RTKs on immune cells and/or tumor cells, and whether and to what extent it occurs should be clarified by using knockout mice of TAM RTKs in future studies.

As professional antigen-presenting cells, cDCs play a critical role in inducing and maintaining antitumor cytotoxic T cell (CTLs) [32]; specifically, a subpopulation of Batf3-dependent CD103^+^ (CD141^+^ in human) cDCs has been defined to be capable of cross-priming antitumor CTLs within lymph nodes and restimulating CTLs within tumors, thus acting as essential initiators and drivers in elicitation of antitumor immunity [28, 33, 34]. CD103^+^ cDCs has been rarely detected and their abundance within tumor tissues is associated with good prognosis and their expansion and activation at tumor sites enhances tumor responses to PD-1 blockade and targeted therapy [33, 35, 36]. Consistent with these studies, we observed that Axl inhibition promoted the accumulation and activation of CD103^+^ cDCs, which may consequently enhance the priming and activation of antitumor CTLs as evidenced by increased effector CD8^+^ T cells; this may also constitute the basis for the synergistic antitumor efficacy of combined Axl inhibition and PD-1 blockade. Future studies are needed to confirm the critical role of these cDCs by using cell depletion experiments or gene-knockout mice and elucidate how Axl inhibition leads to their increased accumulation and activity within tumors.

Our findings provide complementary insights to recent observation that Axl expression is correlated with resistance to ICB-based immunotherapy in mouse tumor models and cancer patients [19, 20]. Thus, through inhibiting the invasion and metastasis of tumor cells and concomitantly eliciting endogenous antitumor immune responses, Axl-targeting therapeutics, alone or optimal combination with other conventional treatments, may overcome the therapeutic resistance and broaden the scope of cancer patients benefited from ICB-based immunotherapy. As several anti-Axl therapeutics are currently being evaluated in clinical trials [22], this proposition has an immediate translational potential, which definitely merits studies in near future. In addition, recent studies have demonstrated that anti-Axl therapeutics, such as Axl-specific antibodies, are able to inhibit tumor growth and metastasis and enhance the therapeutic effect of multiple anticancer therapies (including anti-angiogenesis therapy, targeted therapy and chemotherapy) in the transplantable tumor models or patient-derived xenograft (PDX) models [37–39], indicating a translational potential of combinatorial therapeutics of anti-Axl antibodies and other conventional treatments.

In sum, we demonstrated that Axl inhibition induces antitumor immune responses in two murine tumor models by modulation of CD103^+^ DC accumulation and activation consequently leading to the priming and activation of adaptive antitumor T cells, which reprograms otherwise immunosuppressive microenvironment into a favorable milieu permitting for synergy with PD-1 blockade to elicit a potent antitumor efficacy. Thus, our findings have significant clinical implications as Axl-targeting therapy in Axl expressing tumors could hold a great potential to subvert the innate and/or adaptive resistance to and broaden the coverage of population benefited from ICB-based immunotherapy. Further studies will be needed to more completely define the major biological and immunological effects of Axl inhibition in various settings when we propose combinatorial anti-Axl and ICB-based immunotherapy.

## MATERIALS AND METHODS

### Mice

Six-week-old female C57BL/6 mice, BALB/c mice and BALB/c nude mice were purchased from the Experimental Animal Center of the China Medical University. Animal use was approved by the Institutional Review Board of China Medical University.

### Cell lines

Mouse ID8 ovarian cancer, TC1 lung cancer, 4T1 breast cancer and CT26 colorectal cancer cells were kept in our lab as previously described [40, 41] and routinely screened to be mycoplasma-free. Tumor cells were cultured in the DMEM medium supplemented with 10% FBS (Hyclone), 100 U/mL penicillin and 100 μg/mL streptomycin before cell suspensions were prepared and injected to mice. Isolated lymphocytes were maintained in a complete medium of RPMI-1640 supplemented with 10% FBS, 25 mM HEPES, 2 mM glutamine, 100 U/mL penicillin and 100 μg/mL streptomycin.

### Reagents

Axl tyrosine kinase inhibitors R428 (BGB324) and SGI-7079 were purchased from Selleck Inc. R428 and SGI-7079 specificities for Axl have been previously evaluated [21, 24]. They were dissolved in DMSO at the concentration of 1 mM for *in vitro* studies while formulated in 0.5% hydroxypropylmethylcellulose plus 0.1% Tween 80 for *in vivo* studies. Transfection agent Lipofectamine 2000 was obtained from Invitrogen. Scramble control Axl-targeting siRNA was synthesized by Shanghai GenePharma Inc with the following sequences: siAxl-1: 5′-GCCAGATGAAATCCTCTAT-3′, siAxl-2: 5′-CGAGGTACTTATGGATATA-3′; siNC: 5′-AATTGTACTACACAAAAGTAC-3′. Anti-mouse PD-1 (RMP1–14), CD4 (GK1.5), CD8 (2.43), NK 1.1 (PK136) and their isotype control (2A3) antibodies were purchased from BioXCell. Primary rabbit anti-mouse/human Axl antibody (ab32828) and secondary FITC-labelled goat anti-rabbit antibody (ab6717) were obtained from Abcam. PE-conjugated anti-mouse Axl (FAB8541P), Mer (FAB5912P) and rat IgG2a isotype control (IC006P) antibodies were purchased from R&D Systems. The fluorescence labeled monoclonal antibodies (mAbs) for FACS analysis were all from BD Bioscience.

### Cell proliferation assay

Cells were plated in 96-well plates (5 × 10^3^/well) and then transfected with siNC or siAxl using Lipofectamine 2000 according to the manufacturer's instructions or treated with vehicle, R428 or SGI-7079 at indicated concentrations. After 72 hours of treatment, cell proliferation assays were performed using Cell Counting Kit-8 (Dojindo) according to the manufacturer's instructions. Absorbance was measured at 490 nm using a microplate reader (Molecular devices). The percentage of cell survival was defined as the relative absorbance of untreated versus treated cells.

### Wound healing assay

Cells were plated in 6-well plates (2 × 10^5^/well). Upon 80–90% confluence, the cell layer was scratched with a p-200 pipette tip (1 scratch per well, 3 wells per treatment) and then continued to culture in complete medium in the presence of vehicle, R428 or SGI-7079 at the indicated doses. Alternatively, cells were transfected with siNC or siAxl for 24 hours prior to wound exposure. Photographs of the wound adjacent to reference lines were taken using an Olympus IX51 microscope (10×) at indicated time points. *ImageJ* software was used to digitally measure wound closure; wound closure at each time point was normalized to the averaged scratch width measured at time 0.

### Cell invasion assay

CytoSelect^™^ 24-Well Cell Invasion Assay was purchased from Cell Biolabs Inc. Cells were plated in 24-Well plates in the presence of vehicle, R428 or SGI-7079 at the indicated doses. Alternatively, cells were transfected with siNC or siAxl using Lipofectamine 2000 for 24 hours prior to cell invasion assay. Twenty-four hours post treatment cell invasion was measured as per manufacturer instructions.

### Western blotting and immunofluorescence staining

Whole cell protein lysate was obtained by enhanced RIPA buffer (Beyotime). Samples were sonicated and then centrifuged at 15,000 g for 10 min at 4°C. Protein concentrations were determined by Bradford assay (Beyotime). Equal amounts of protein were fractionated by SDS-PAGE, transferred to a PVDF membrane (Millipore), and analyzed by incubation with primary rabbit anti-mouse/human Axl antibody. Proteins were detected via incubation with HRP-conjugated secondary antibodies and enhanced chemiluminescence (ECL) detection system (Cell Signaling Technology).

For immunofluorescence staining, tumor cells were seeded on collagen-coated glass slides and grown to 50–60% confluence. Cells were then fixed in fresh 3% formaldehyde in PBS followed by permeabilization and blocking in Tris-buffered saline + 0.05% Tween 20 (TBST) containing 5% bovine serum albumin and 0.3% Triton X-100 at room temperature. After that, cells were stained with primary rabbit anti-mouse/human Axl antibody followed by secondary FITC-labeled goat anti-rabbit antibody. After final washes in TBST, cells were mounted in ProLong Gold anti-fade reagent (Life Technologies) and imaged on the Olympus FV1000 confocal microscope system. Acquired images were processed by ImageJ software.

### Tumor challenge and treatment experiments

BALB/c syngeneic mice or nude mice (6 mice per group) were inoculated subcutaneously with 5 × 10^5^ 4T1 cells in 100 μl of PBS while C57BL/6 syngeneic mice or nude mice (6 mice per group) were inoculated intraperitoneally (i.p.) with 1 × 10^6^ ID8 cells in 100 μl of PBS. After 7 or 10 days, animals received either R428, SGI-7079, anti-PD-1 mAb alone or combined treatment. R428 or SGI-7079 was orally administered at indicated doses 5 days per week for 2 weeks while anti-PD-1 mAb was i.p. injected twice per week for 2 weeks. Mice treated with vehicle and/or control antibody (2A3) were used as controls. Treated BALB/c mice were monitored for tumor growth and body weight measurements twice weekly. Mice were euthanatized when they seemed moribund or their tumors reached 15 mm in diameter. Tumor volumes were measured by caliper and calculated using the standard formula 1/2((Length × Width) 2). Treated C57BL/6 mice were monitored daily for the signs of ascites formation with body weight twice per week. When mice developed ascites and had a weight increase > 30%, they were euthanized at which time was recorded and defined as dead time point.

For *in vivo* lymphocyte depletion experiments, mice received the following mAbs via i.p. injections: anti-CD8 (clone 2.43; 200 μg per injection); anti-CD4 (clone GK1.5; 200 μg per injection); or anti-NK1.1 (clone PK136; 300 μg per injection) on day −3, −1, +4, +10 and +17 after first treatment. Depletions were confirmed by FACS analysis of blood samples (data not shown).

For rechallenge experiments, long-term survivors (LTS) from first tumor challenge were rechallenged with 1 × 10^5^ of ID8 (i.p.) or TC1 (s.c.) tumor cells and their overall survival was evaluated. Naïve mice with same challenge were used as controls.

### FACS analysis

Mice bearing ID8 or 4T1 tumors established as described above were treated with vehicle, R428 (100 mg/kg) or SGI-7079 (50 mg/kg) for the consecutive 5 days, and 2 days later, tumors were dissected, cut into small pieces and digested with Collagenase IV (200 U/ml) and DNase I (0.2 mg/ml) for one hour at 37°C. After digestion, tumors were passed through 70-μm filters and washed with FACS buffer (PBS containing 2% FBS, 2 mM EDTA and 0.02% sodium azide) and pelleted. Single-cell suspensions were then preincubated with mouse Fc receptor blocker (clone 24G2) for 10 minutes before staining with mAbs specific for CD45 (30-F11), CD3 (145-2C11), CD4 (RM4-4), CD8 (53–6.7), CD11b (M1/70), F4/80 (BM8), Gr-1 (RB6-8C5), CD49b (DX5), CD69 (H1.2F3), CD40 (MR1), CD86 (GL-1), MHC-II (M5/114.15.2), CD11c (HL3), CD103 (M290), PD-1 (J43), PD-L1 (MIH5) and MHC-I (AF6-88.5) for 30 minutes at 4℃ followed by 2 washes in FACS buffer. For Ki67 and FoxP3 staining, cells were fixed by using the Cytofix/Cytoperm Kit after surface marker staining and then stained with anti-mouse/human Ki-67 (B56) and anti-FoxP3 (236A/E7) mAbs. *Ex vivo* intracellular IFN-γ and IL-12 p40 stainings were performed on isolated cells 6 hours following an intravenous injection of Brefeldin A (10 mg/g of body weight, Sigma-Aldrich). Tumor digestion was performed in the presence of Brefeldin A (5 μg/ml). Cells were then stained for surface markers, fixed, permeabilized and then stained with anti-mouse IFN-γ (clone XMG1.2) and IL-12/23 p40 mAbs (clone C15.6). Propidium iodide or 7-Amino-Actinomycin D were added to the final suspension to exclude dead cells before acquisition on a FACS Calibur (BD Biosciences) and analysis performed with Flowjo software. Live cell counts were calculated from the acquisition of a fixed number of 10 μm latex beads (Coulter) mixed with a known volume of unstained cell suspension.

For the detection of mouse Axl or Mer expression by FACS, mouse tumor cells was stained with PE-conjugated anti-mouse Axl or Mer antibody (R&D Systems) with appropriate isotype antibody as control.

### qPCR analysis

Total RNA of tumors isolated from treated mice was extracted using RNeasy Mini Kits (Qiagen) and reverse transcribed into cDNA using SuperScript III Reverse Transcriptase (Invitrogen) according to the manufacturer's instructions, and then qPCR was performed as previously described [41]. The primers for all genes tested, including internal control GAPDH, were synthesized by Takara Inc. Primer sequences were shown in Supplementary Table 1. qPCR was performed via ABI PRISM 7500 Real-Time PCR Systerm (Applied Biosystems) with 1 × SYBR Green Universal PCR Mastermix (Takara). Transcript levels were calculated according to the 2–ΔΔCt method, normalized to the expression of GAPDH, and expressed as fold change compared with control.

### Statistics

All analyses and graphics were done using GraphPad Prism 5 (GraphPad Software). All data are expressed as mean ± SEM. Differences between groups were evaluated by two-tailed Student's *t* test or one-way ANOVA followed by Tukey's multiple comparisons test; Survival rates were analyzed using the Kaplan–Meier method and evaluated with the log-rank test. Significant differences were accepted at *p* < 0.05.

## SUPPLEMENTARY MATERIALS FIGURES AND TABLE


