# Supplementary material for: Axl inhibition induces the antitumor immune response which can be further potentiated by PD-1 blockade in the mouse cancer models
Source: Oncotarget. 2017 Sep 21;8(52):89761–74. doi: 10.18632/oncotarget.21125 (PMC5685707; doi:10.18632/oncotarget.21125)
Supplement: Supplementary file 1 [file oncotarget-08-89761-s001.pdf]

Axl inhibition induces the antitumor immune response which can be further potentiated by PD-1 blockade in the mouse cancer models

SUPPLEMENTARY MATERIALS

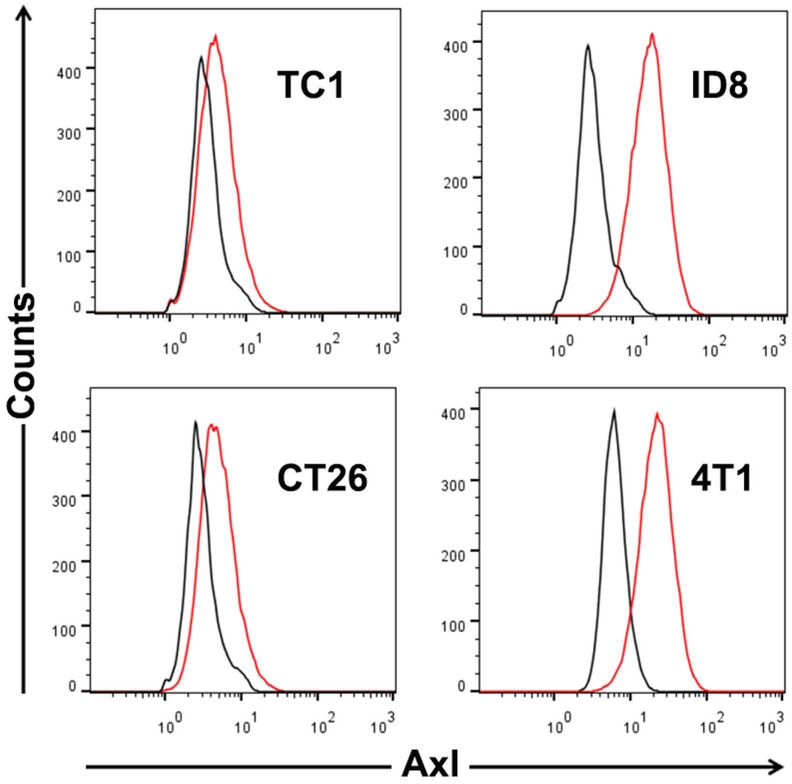

Supplementary Figure 1: The representative FACS plots for Axl expression in 4 mouse tumor cells. Black and red line denotes the isotype control and Axl staining respectively.

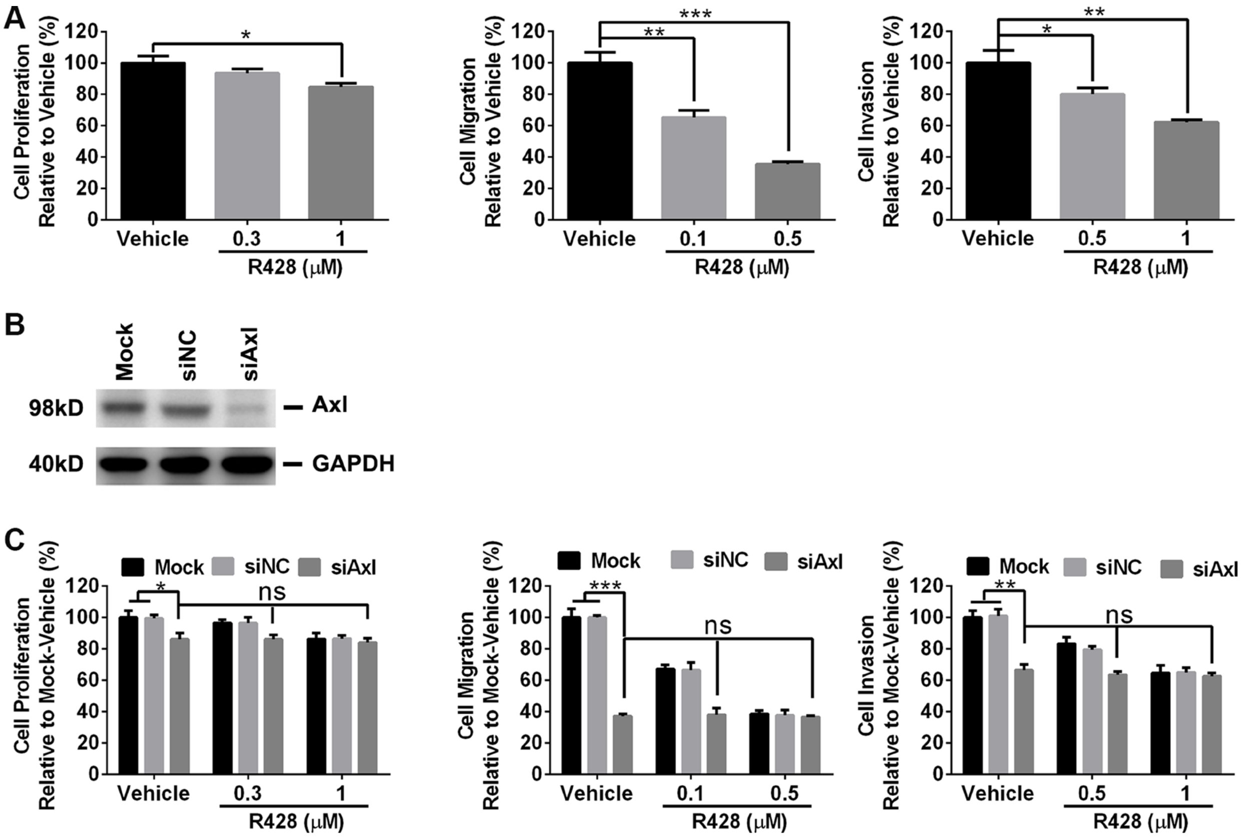

Supplementary Figure 2: (**A**) 4T1 cells were treated with vehicle or R428 at indicated doses before performing proliferation, migration and invasion assays. (**B–C**) 4T1 cells were transfected with mock, control siNC or siRNA specific for Axl (siAxl-2) and subsequently subjected to western blotting for confirmation of Axl knockdown (B) or proliferation, migration and invasion assays (C). Proliferation and invasion was plotted as a percentage of growth relative to vehicle-treated cells with migration plotted as a percentage relative to zero time point of each treated cells. The experiments were performed thrice with 3 technical replicates, and data are expressed as mean ± SEM, ns, not significant, *$p < 0.05$, **$p < 0.01$, ***$p < 0.001$, one-way ANOVA followed by Tukey's multiple comparisons test.

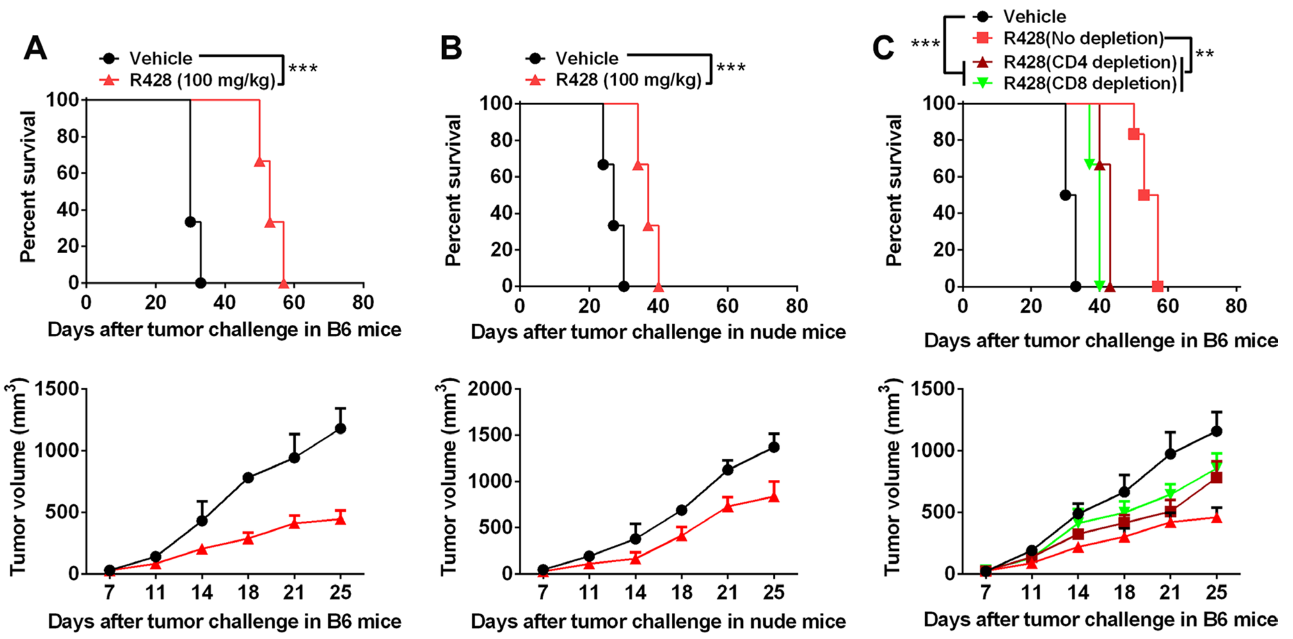

Supplementary Figure 3: BALB/c mice (**A**) or nude mice (**B**; all 6 per group) bearing 7-day-established 4T1 tumors were treated with R428 at the indicated dose for two weeks and their overall survival (top) and tumor growth (bottom) was evaluated. (**C**) BALB/c mice (6 per group) bearing 7-day-established 4T1 tumors with lymphocyte subset depletion were treated with R428 at the indicated dose for two weeks and their overall survival (top) and tumor growth (bottom) was evaluated. Data are representative of two independent experiments, $^{**}p < 0.01$, $^{***}p < 0.001$, log-rank test.

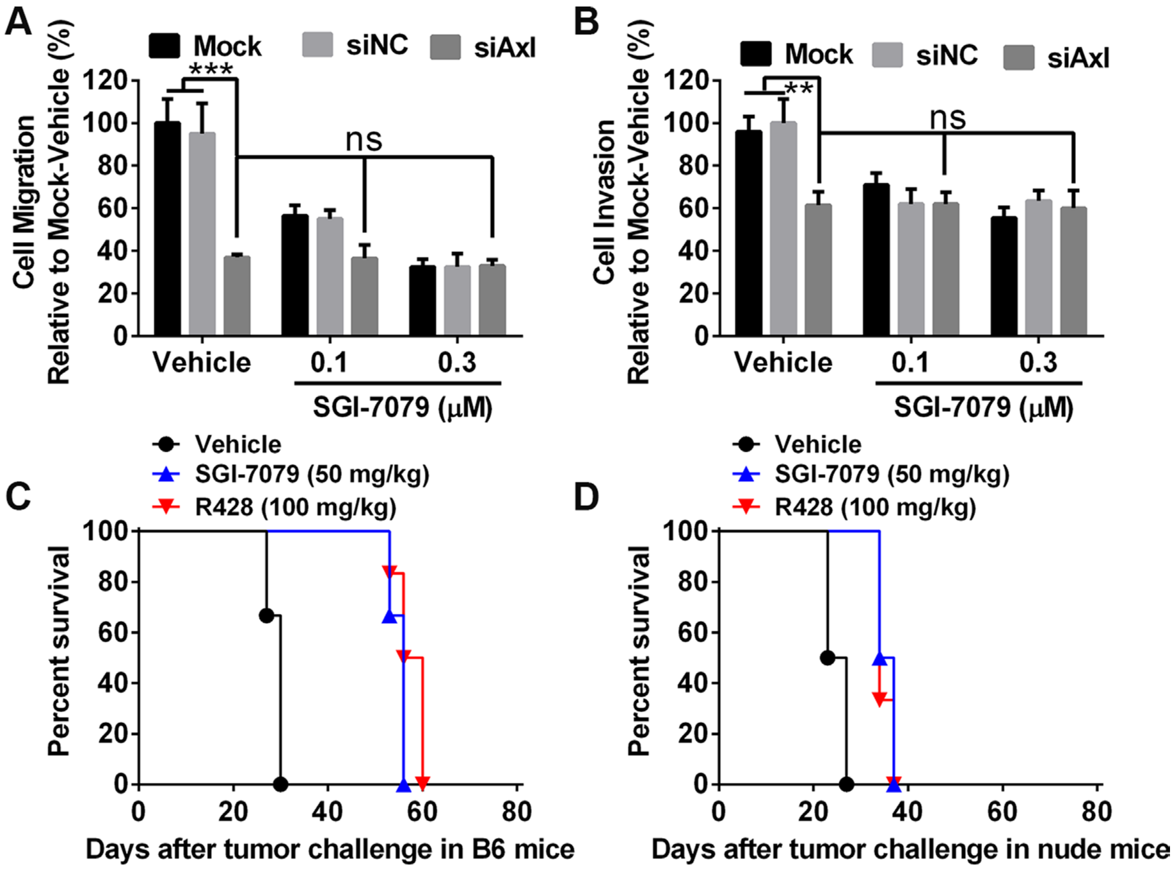

Supplementary Figure 4: (**A**–**B**) ID8 cells were transfected with mock, control siNC or siRNA specific for Axl (siAxl-2) and subsequently subjected to migration (A) and invasion assays (B). Migration and invasion was plotted as a percentage relative to vehicle-treated mock cells. (C–D) C57BL/6 mice (**C**) or nude mice (**D**) bearing 10-day-established ID8 tumors were treated with control vehicle, R428 or SGI-7079 at the indicated dose for two weeks and their overall survival was evaluated. The experiments were performed twice with 3 technical replicates, and data are expressed as mean ± SEM (for A and B) and are representative of two independent experiments (C and D), ns, not significant, $^{**}p < 0.01$, $^{***}p < 0.001$, one-way ANOVA followed by Tukey's multiple comparisons test.

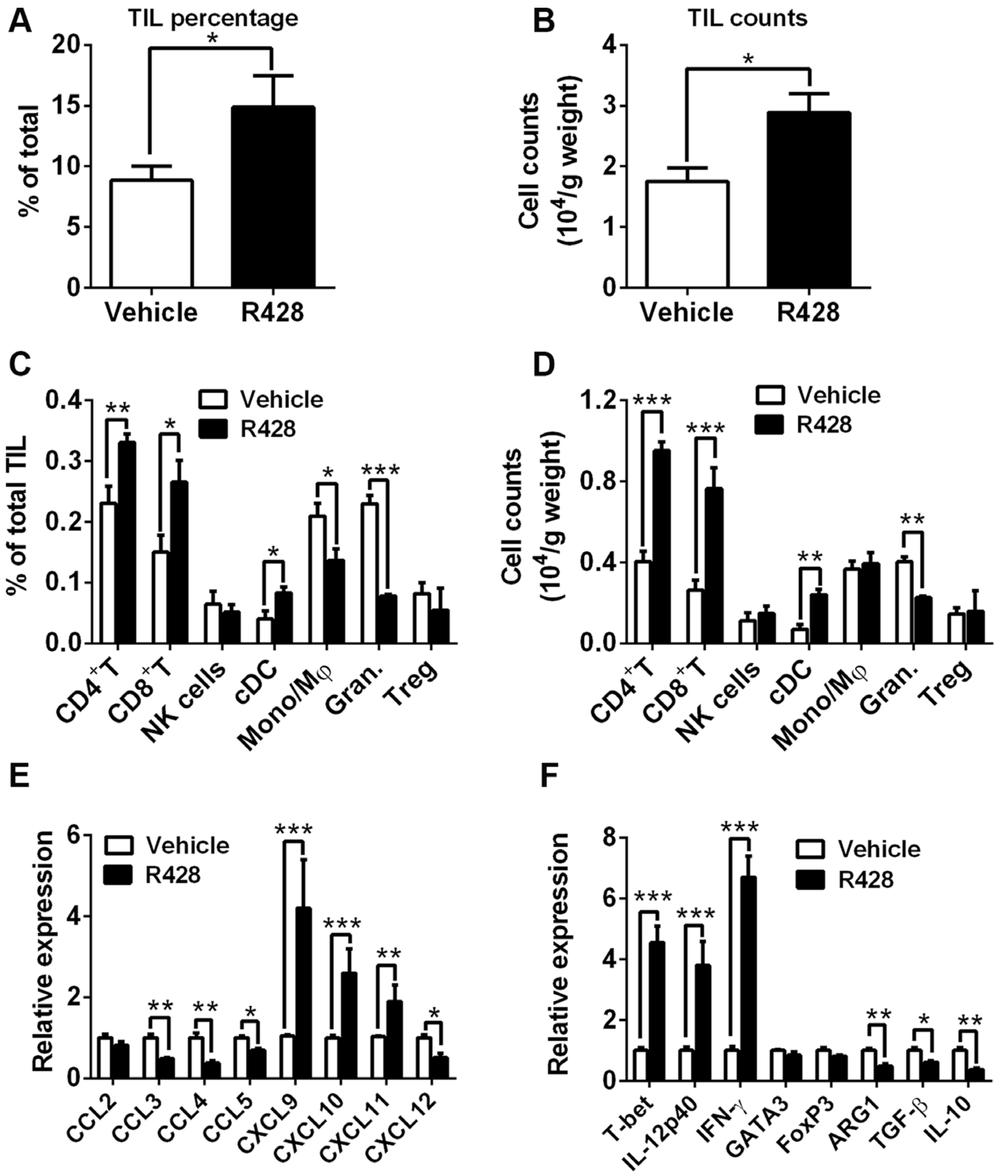

Supplementary Figure 5: BALB/c mice (3 per group) bearing 7-day-established 4T1 tumors were treated with control vehicle or R428 for 5 days and then TILs and immune-associated genes within tumors were analyzed by FACS and qPCR respectively. (**A–B**) The percentage (A) and absolute number (B) of $CD45^+$ TILs in tumors from vehicle or R428 treated mice. (**C–D**) The percentage (C) and absolute number (D) of $CD4^+$ $FoxP3^-$ T cells, $CD8^+$ T cells, $CD3^+$ $NK1.1^+$ NK cells, $CD11c^+$ $MHCII^+$ conventional DCs (cDCs), $CD11b^+$ $F4/80^+$ $Ly\text{-}6G^-$ monocytes/macrophages (Mono/Mφ), $CD11b^+$ $F4/80^-$ $Ly\text{-}6G^+$ granulocytes and $CD4^+FoxP3^+$ regulatory T (Treg) cells within TILs. (**E–F**) The expression of genes associated with type-1 T cell or suppressive cells recruitment (E) and functionality (F). Mean values of messenger RNA levels in control group were set to 1. Data are derived from three tumors per treatment and representative of two independent experiments, $*p < 0.05$, $**p < 0.01$, $***p < 0.001$, two-tailed student *t* test.

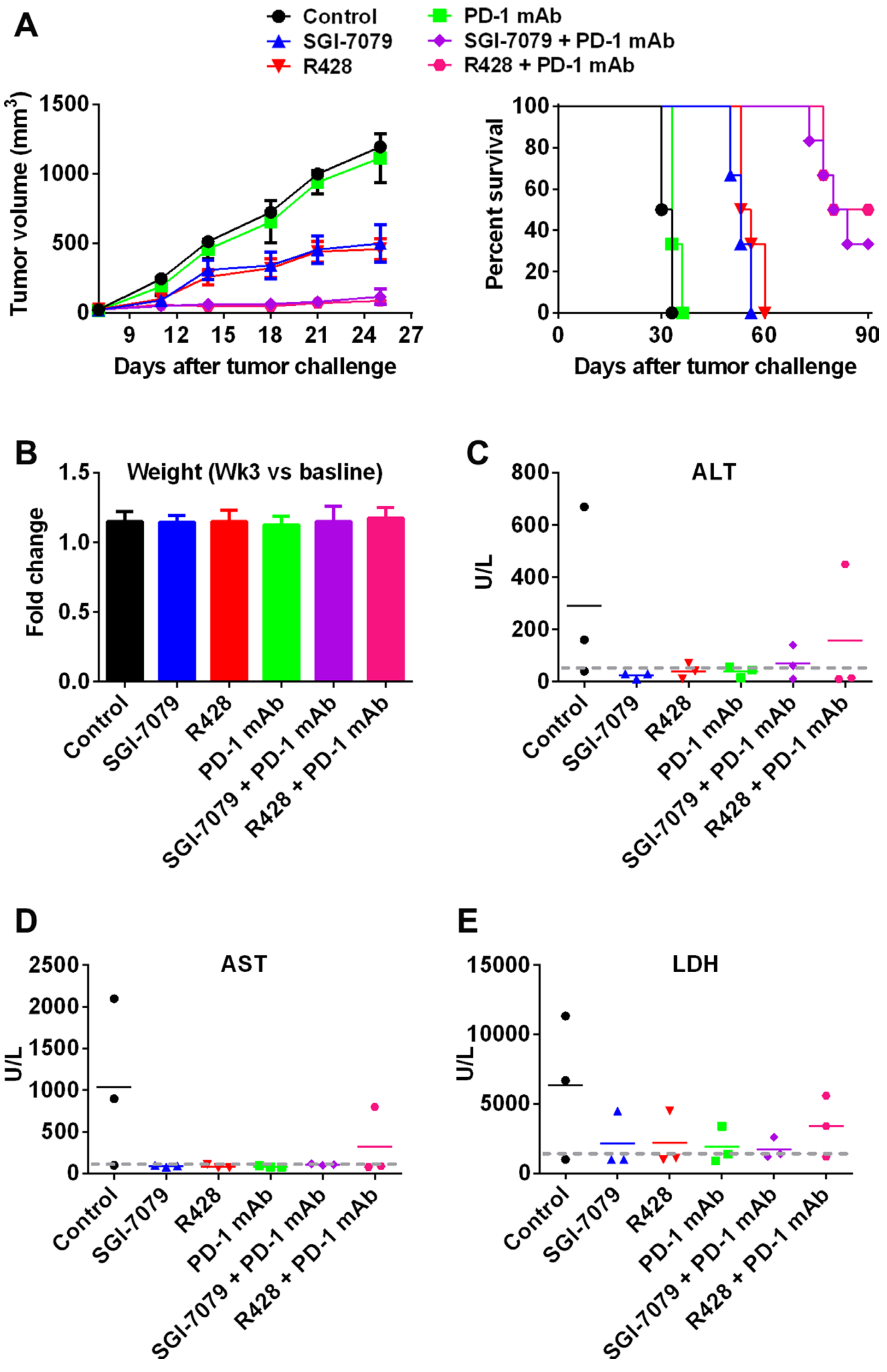

Supplementary Figure 6: (**A**) BALB/c mice (6 per group) bearing 7-day-established 4T1 tumors were treated with either single or combined R428/SGI-7079 and anti-PD-1 mAb for two weeks as scheduled in Fig.6C and tumor growth (left) and their overall survival (right) was evaluated. (**B**–**E**) BALB/c mice (3 per group) bearing 7-day-established 4T1 tumors were treated with either single or combined R428/SGI-7079 and anti-PD-1 mAb for two weeks as scheduled in Fig.6C and general health status and weight of treated mice were monitored during the experiment. Three weeks after the start of the treatment, mice were euthanized for tissue analysis. Fold change of total body weights at baseline versus at the end of the experiment and liver enzymes (AST, aspartate aminotransferase; ALT, alanine aminotransferase) and lactate dehydrogenase (LDH) levels in serum at the end of the experiment. The dotted line indicates the upper limit of normal reference values.

Supplementary Table 1: Primers used in real-time PCR

GAPDH	Sense:5′-GTGGAGATTGTTGCCATCAACG-3′<br>Antisense:5′-CAGTGGATGCAGGGATGATGTTCTG-3′
CCL2	Sense:5′- CAATGAGTAGGCTGGAGAGC-3′<br>Antisense: 5′-TGAAGACCTTAGGGCAGATG-3′
CCL3	Sense: 5′-TGAAACCAGCAGCCTTTG-3′<br>Antisense: 5′-CCAGGTCAGTGATGTATTCTTG-3′
CCL4	Sense: 5′-TCTGCCCTCTCTCTCCTCTT -3′<br>Antisense: 5′-CTGCTGGTCTCATAGTAATCCA-3′
CCL5	Sense: 5′-TGCTGCTTTGCCTACCTCT-3′<br>Antisense: 5′-ACACACTTGGCGGTTCCTT-3′
CXCL9	Sense:5′-TTTTGGGCATCATCTTCCTGG-3′<br>Antisense: 5′-GAGGTCTTTGAGGGATTTGTAGTGG-3′
CXCL10	Sense:5′-CTTCTGAAAGGTGACCAGCC-3′<br>Antisense: 5′-GTCGCACCTCCACATAGCTT-3′
CXCL11	Sense:5′- AGGAAGGTCACAGCCATAGC-3′<br>Antisense: 5′- AACTTTGTCGCAGCCGTT-3′
CXCL12	Sense:5′-CCTCAACACTCCAAACTGT -3′<br>Antisense: 5′-CTTTCTCTTCTTCTGTCGCTTC-3′
T-bet	Sense: 5′-CGGTACCAGAGCGGCAAGT-3′<br>Antisense: 5′-AGCCCCCTTGTTGTTGGTG-3′
IL-12p40	Sense:5′-ACATCACCTGGACCTCAGAC -3′<br>Antisense: 5′- TTCCTTCTTGTGGAGCAGC-3′
IFN-γ	Sense: 5′-AAAAACCTAAAAAATCTAAATAACT-3′<br>Antisense: 5′-ATCAACAACAACTCCTTTTCCACTT-3′
GATA3	Sense: 5′-ACTCCAGTCCTCATCTCTTCA-3′<br>Antisense: 5′-GCACTCTTTCTCATCTTGCC-3′
FoxP3	Sense:5′-CAGCTGCCTACAGTGCCCCTAG-3′<br>Antisense:5′-CATTTGCCAGCAGTGGGTAG-3′
ARG1	Sense:5′- GAAGAATGGAAGAGTCAGTGTG-3′<br>Antisense: 5′- TGTTGATGTCAGTGTGAGCA-3′
TGF-β1	Sense:5′-GTGGTATACTGAGACACCTTGG-3′<br>Antisense:5′-CCTTAGTTTGGACAGGATCTGG-3′
IL-10	Sense:5′-CTCTTACTGACTGGCATGAGG-3′<br>Antisense:5′-CCTTGTAGACACCTTGGTCTTGGAG-3′
